# Extending the Grazing Period for Bulls, Prior to Finishing on a Concentrate Ration: Composition, Collagen Structure and Organoleptic Characteristics of Beef

**DOI:** 10.3390/foods8070278

**Published:** 2019-07-23

**Authors:** Gebrehawerya B. Mezgebo, Frank J. Monahan, Mark McGee, Edward G. O’Riordan, Declan Marren, Anne Listrat, Brigitte Picard, R. Ian Richardson, Aidan P. Moloney

**Affiliations:** 1 School of Agriculture and Food Science, University College Dublin, D04V1W8 Dublin, Ireland; 2 Teagasc, Animal & Grassland Research and Innovation Centre, Grange, Dunsany, Co., C15PW93 Meath, Ireland; 3 UMR1213 Herbivores, INRA, VetAgro Sup, Clermont Université, Université de Lyon, F-63122 Saint-Genès-Champanelle, France; 4 Division of Farm Animal Science, Department of Clinical Veterinary Medicine, University of Bristol, Langford, Bristol BS40 5DU, UK

**Keywords:** beef, fat score, intramuscular fat, fibre type, sensory quality

## Abstract

The biochemical and organoleptic characteristics of the longissimus thoracis muscle from suckler bulls (*n* = 56) finished on a concentrate-based system (C) or raised in a pasture-based system (P) incorporating 99 (P99), 162 (P162) or 231 days (P231) of grazing prior to indoor finishing on the concentrate-based diet were investigated. Age at slaughter increased with increasing period at pasture. Intramuscular fat concentration was lower (*p* < 0.001) for P99 than for C, P162 and P231 bulls, which did not differ. Soluble collagen proportion was lower (*p* < 0.01) for P162 and P231 than for P99 and C bulls. Collagen cross-link content was higher (*p* < 0.05) for P231 than for P99 and C bulls and for P162 than for C bulls. The proportion of type I muscle fibres was higher (*p* < 0.01) for P231 and P162 than for P99 and C bulls. Sensory tenderness was higher (*p* < 0.001) for C and P162 than for P99 and P231 bulls and overall liking was higher (*p* < 0.01) for C than for P99 and P231 bulls but similar to P162 bulls. Extending the grazing period to 162 days did not negatively influence the sensory qualities of beef compared to the intensive concentrate-based system.

## 1. Introduction

Traditionally, suckler bull beef production was based on the provision of concentrate-based diets until slaughter (i.e., intensive indoor systems) [1,2]. The profitability of such systems in temperate regions, can be enhanced by the use of cheaper feedstuffs such as conserved or grazed pasture [3]. Thus, O’Riordan et al. [2] showed that introducing a pasture grazing period of 100 days for suckler bulls prior to finishing on concentrates decreased the cost of production. It was also reported that in such suckler beef systems, slaughtering bulls at under 20 months of age was desirable, as some markets have an age restriction [1]. However, suckler bulls from such systems, which usually involve late maturing breed types, risked not achieving the desired carcass fat classification [2,4]. Similarly, such modifications (i.e., incorporating a grazing period) can affect the compositional and eating quality of beef [4]. 

The longer bulls are at pasture, the cheaper the cost of production. However, the trade-off between the duration of grazing, carcass fat classification and the extent of the concentrate feeding during finishing is important economically. There is a need therefore to investigate the effect of extending the grazing period combined with finishing suckler bulls on a cereal concentrate on carcass fat classification, as well as other important carcass and meat quality traits. In addition to maximising economic returns, beef from grass fed animals is favoured by some consumers, as it is considered more ‘natural’ [5] and reported to enhance the proportion of nutritionally important fatty acids [6,7,8]. However, the provision of grass for a prolonged period in bull beef production systems would likely increase the age at slaughter, which may influence consumer preferences and the sensory qualities of beef [9,10].

Therefore, the objective of the study was to examine the effect of varying the grazing period from 0 to approximately 8 months prior to finishing on a cereal-based concentrate (to achieve a carcass fat classification similar to that achieved by the intensively concentrate fed bulls) on fat and muscle colour, biochemical and sensory characteristics of beef. It was hypothesized that extending the grazing period up to a full season and its associated increase in slaughter age would negatively influence the quality of beef, particularly sensory tenderness.

## 2. Materials and Methods

### 2.1. Animals and Management

Fifty-six spring born (mean birth date 8 March) Charolais and Limousin sired weaned suckler bulls were purchased at livestock marts in Ireland at approximately 8 months of age and an average initial weight of 372 kg. They were acclimatised to slatted floor accommodation and offered a predominantly perennial grass silage ad libitum plus 2 kg/head/day of a barley-based concentrate before random assignment (1 December) to four treatments (14 animals per treatment) balanced for sire breed, birth date and initial weight. The four treatments were: (1) ad libitum concentrates (870 g/kg rolled barley, 60 g/kg soya bean meal, 50 g/kg molasses and 20 g/kg minerals/vitamins) plus grass silage (dry matter (DM) digestibility 700 g/kg) ad libitum until slaughter (C), (2) grass silage ad libitum plus 2 kg concentrate daily during the winter (123 days duration) (GS) followed by 99 days at pasture (predominantly perennial ryegrass) and then an indoor finishing period on C until slaughter (P99), (3) GS followed by 162 days at pasture and then an indoor finishing period on C until slaughter (P162) and (4) GS followed by 231 days at pasture and then an indoor finishing period on C until slaughter (P231). The mean age and live weight were 267 days (sd = 29.0) and 377 kg (sd = 33.1), respectively. A rotational grazing management strategy was used when animals were at pasture. The duration of the concentrate finishing periods were 201, 71, 120 and 121 days for C, P99, P162 and P231 bulls, respectively. The strategy was for C and P99 to have similar carcass weight and for C, P162 and P231 to have a similar carcass fat score. The study was carried out under license from the Irish Government Department of Health and Children and with the approval of Teagasc, the Agricultural and Food Development Authority. All procedures used complied with national regulations concerning experimentation on farm animals.

### 2.2. Carcass Grading and Muscle Tissue Collection

On the day of slaughter, bulls were transported approximately 30 km to a commercial slaughter plant (Kepak Group, Clonee, Co., Meath, Ireland) and slaughtered immediately after arrival by bolt stunning followed by exsanguination from the jugular vein. Electrical stimulation was not applied and carcasses were hanged by the Achilles tendon. The slaughter and dressing procedures were in accordance with the Regulations (EC) No. 1099/2009 and No. 853/2004. Post slaughter, carcasses were weighed and graded for conformation (15 point scale, classes E^+^ (highest) to P^−^ (lowest), E^+^ is 15) and fatness (15 point scale, scores 5^+^ (highest) to 1^−^ (lowest), 5^+^ is 15) according to the EU Beef Carcass Classification Scheme [11]. Approximately 45 min after slaughter, carcasses were placed in a chill set at 9 °C and ambient temperature was monitored (2 h = 6.6 °C, 3.5 h = 7.6 °C, 5 h = 8.1 °C). After approximately 10 h the chill temperature was reduced to 0 °C. At 1 h post-slaughter, a sample (ca. 20 g) of longissimus thoracis (LT) muscle tissue (from the 9th rib position) was taken, snap frozen in liquid nitrogen and maintained at −80 °C for metabolic enzyme activity and fibre typing analyses.

### 2.3. Muscle pH and Temperature Measurement

Muscle pH was measured at 2, 3.5, 5 and 48 h post-mortem by making a scalpel incision in the muscle at the 10th rib and inserting a glass electrode (Model EC-2010-06, Reflex Sensors Ltd., Westport, Co., Mayo, Ireland) attached to a portable pH meter (Model no. 250A, Orion Research Inc., Boston, MA, USA) approximately 4.0 cm into the muscle. The temperature was recorded simultaneously and used to make a temperature compensated pH measurement.

### 2.4. Fat and Muscle Colour Measurements

A detailed procedure is given in [4]. Briefly, at 48 h post-mortem, carcasses were cut at the 5/6 rib interface prior to subcutaneous fat and muscle colour measurements. Subcutaneous fat colour was measured using a Miniscan XE Plus (Hunter Associates Laboratory Inc., Reston, VA, USA) at two positions: (1) the surface of the lower round/rump region and (2) the surface of the 13th rib region. For muscle colour measurement, the cut surface of the muscle was first allowed to bloom for 1 h and the colour (L, a, b) was then measured using the Miniscan XE Plus. The L, a, b colour coordinates represent lightness (scale 0 (black) to 100 (white)), redness (scale +a (red) to –a (green)) and yellowness (scale +b (yellow) to −b (blue)) of the muscle respectively. A chroma (saturation) colour intensity, C (computed as √(a^2^ + b^2^), where a higher ‘*C*’ value indicates higher colour saturation) and hue angle, H (computed as [tan^−1^(b/a)][180/π], where 0/360° is red, 90° is yellow, 180° is green and 270° is blue colour) were also determined. Muscle pH and temperature were measured as described above. The cube roll (commercial cut that begins between the 5th and 6th rib and ends between the 10th and 11th rib) was then removed, The LT perimeter was drawn on translucent paper and the area was subsequently measured using a digital planimeter (Koizumi Placom, KP-90N, Niigata, Japan). The ribs joint was weighed, dissected into the LT, other lean muscle, fat and bone and the components weighed. The LT from the cube roll was vacuum packed, transported to Teagasc, Food Research Centre, Ashtown, Dublin, aged for 14 days at 2 °C and then stored at −18 °C prior to composition, collagen solubility and cross-link, proteoglycan and sensory analyses.

### 2.5. Proximate Composition, Collagen Content and Sensory Analyses

Moisture, intramuscular fat (IMF) and protein concentration of the LT muscle were determined using the SMART System 5 microwave moisture drying oven, NMR SMART Trac rapid fat analyser (CEM Corporation, Matthews, NC, USA) and LECO FP328 (LECO Corporation, Joseph, MI, USA) protein analyser, respectively [12]. Ash was determined by incinerating samples in a furnace (540 °C overnight). Collagen content was determined by quantitative determination of hydroxyproline by a colorimetric reaction [13]. Sensory analysis was conducted at the University of Bristol using a 10-person trained taste panel who had been selected for their sensory acuity using the methods outlined in BSI [14]. Panellists tasted the samples from every animal in an order based on the designs outlined in [15] for balancing carryover effects between samples. The panellists assessed each steak using 0–100 mm unstructured intensity line scales for a consensually agreed texture profile, where 0 = nil and 100 = extreme, and 8 point category scales for tenderness (1 = extremely tough to 8 = extremely tender), juiciness (1 = extremely dry to 8 = extremely juicy), beefy flavour and abnormal beef flavour intensities (1 = extremely weak to 8 = extremely strong). A detailed description of the compositional, collagen and sensory analyses methods is given in [4].

### 2.6. Collagen Cross-Link Analysis

Collagen cross-links were determined as previously described [16]. Briefly, frozen muscle samples were cut into 1 cm^3^ cubes and powdered in liquid nitrogen prior to analysis. For collagen cross-link determination, about 250 mg of muscle powder were weighed and hydrolysed with 10 mL of 6 N HCl overnight at 110 °C in a screw-capped glass tube. After hydrolysis, 1 mL of the acid hydrolysate was centrifuged at 16,000× *g* for 5 min at 4 °C. Then 600 μL of 6 N NaOH and 600 μL of 1 M Tris-HCl were added to 600 μL of acid supernatant. Final pH was checked using pH test strip and adjusted to between 7 and 7.5 using 6 N HCl or NaOH. Pyridinoline cross-links (pyridinoline + deoxypyridinoline) were determined by the enzyme-linked immunoassay Metra Pyd EIA kit (Quidel Corporation, San Diego, CA, USA). Results are expressed in nM of pyridinoline per g DM.

A detailed description of the proteoglycan determination method is given in [16]. Briefly, 100 mg muscle powder were incubated for 24 h at 4 °C with 1 mL extraction buffer (6 M Urea, 1 M NaCl, 2% CHAPS (3-((3-Cholamidopropyl) dimethylammonio)-1-propanesulfonate hydrate) and protease inhibitor cocktail (1 tablet per 50 mL) (Roche Diagnostics GmbH, Mannheim, Germany). Following centrifugation (40 min at 4 °C, 15,000× *g*) the supernatant was recovered and used to determine proteoglycan content [17,18]. This assay is based on the ability of sulphated glycosaminoglycans (GAGs) to bind the cationic dye 1, 9-dimethylmethylene blue (DMMB). Briefly, 1 mL of DMMB solution was added to 100 μL of muscle extract and shaken for 30 min at room temperature. After centrifugation for 15 min at 12,000× *g*, the supernatant (containing DMMB excess) was removed. One ml of 50 mM sodium acetate buffer solution was added to the residue and shaken for 30 min. Absorbance was measured at 656 nm, with a micro-plate reader (TECAN Infinite, M200, Lyon, France). Concentrations were determined by comparison with a standard curve of chondroitin-4-sulfate (C4S) in the range 0 to 2.5 μg/mL. The data are expressed in μg of C4S-GAGs equivalents per g of DM.

### 2.7. Muscle Metabolic Enzyme Activity and Fibre Typing

Glycolytic enzyme activities (lactate dehydrogenase (LDH) and phosphofructokinase (PFK)) and oxidative enzyme activities (isocitrate dehydrogenase (ICDH), citrate synthase (CS) and cytochrome *c* oxidase (COX)) were quantified spectrophotometrically [19]. Muscle fibre types were analysed using high-resolution mini-gel electrophoresis [20].

### 2.8. Statistical Analysis

Data were subjected to analysis of variance using the General Linear Model procedure of SPSS (IBM SPSS Statistics Version 20) where the production system was regarded as a fixed factor. Assessor and session were also regarded as fixed factors when analysing the sensory data. Means were considered significant at *p* < 0.05. Muscle pH and temperature data were analysed using the GLM repeated measures (GLM REP) procedure of SPSS. Data were also subjected to multiple analysis of variance to calculate partial correlation coefficients (*p*), from the error sum of squares and cross products (SSCP) matrix, between selected production and carcass traits, muscle biochemical composition and sensory qualities of beef.

## 3. Results

### 3.1. Production and Carcass Traits

**Figure 1:**
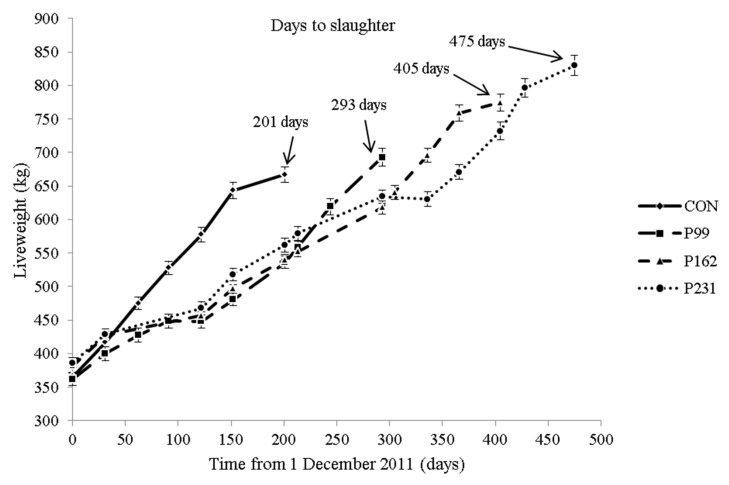
Growth pattern of bulls raised on either a concentrate-based system (C) or pasture-based systems (P) incorporating 99 days (P1), 162 days (P2) or 231 days (P3) of grazing period prior to indoor finishing on a concentrate-based diet (*n* = 14/treatment).

The growth pattern of the bulls is shown in Figure 1. 

Considering 1 December as day 1 of the experiment, all grazing bulls were turned out to pasture on day 123. P99, P162 and P231 bulls were re-housed on day 222, 285 and 354, respectively. Data on production and carcass traits are presented in Table 1. 

Age at slaughter was lower (*p* < 0.001) for C than for P99, for P99 than for P162 and for P162 than for P231 bulls. The average daily liveweight gain (ADG) during finishing was higher (*p* < 0.001) for P99 than for P162, P231 and C bulls; for P231 than for P162 bulls, but similar for C and for P162, and for C and for P231 bulls. The ADG overall was higher (*p* < 0.001) for C than for P99 bulls, which in turn was higher than for P162 and P231 bulls, which did not differ. Slaughter and carcass weights were lower (*p* < 0.001) for C and P99 (which did not differ) than for P162 bulls, which in turn was lower (*p* < 0.001) than for P231 bulls. Fat score was lower (*p* < 0.001) for P99 than for P162, P231 and C bulls, which did not differ. Of the subcutaneous fat colour coordinates, ‘L’ value was lower (*p* < 0.001) for P99 than for P162, P231 and C bulls, which did not differ. The weight of the cube roll was similar for C and P99 and lower (*p* < 0.001) for P162 and P231 which did not differ. The weight of the LT within the cube roll was lower (*p* < 0.05) for C than for P162, but P99, P162 and P231 did not differ. The cross-sectional area of the LT at the 10th rib was lower for C than for P99, P162 and P231, which did not differ. The proportion of fat was lower and the proportion of lean was higher (*p* < 0.05) for C compared to P99, P162 and P231, which did not differ.

### 3.2. Muscle pH and Temperature Profile

Early post-mortem muscle pH and temperature data are presented in Table 2. 

There was an interaction (*p* < 0.001) between production system and time post-mortem with respect to muscle pH. Thus, at 2 h post-mortem muscle pH was higher (*p* < 0.01) for C and P231 (which did not differ) than for P99 and P162 which did not differ. At 3.5 h post-mortem muscle pH was higher (*p* < 0.001) for C, P99 and P231 (which did not differ) than for P162. At 5 h post-mortem, muscle pH was similar for C, P99 and P231, lower (*p* < 0.05) for P162 than for C and P99 but similar to P231. 

There was an interaction (*p* < 0.001) between production system and time post-mortem with respect to muscle temperature. Thus, at 2 h post-mortem, muscle temperature was lower (*p* < 0.001) for P99 than for C and P162 (which did not differ), which in turn was lower than for P231. At 3.5 h post-mortem, muscle temperature was lower (*p* < 0.001) for C and P99 (which did not differ) than for P162, which in turn was lower than for P231. At 5 h post-mortem, muscle temperature was lower (*p* < 0.001) for P99 than for C, P162 and P231, which did not differ. 

### 3.3. Muscle Colour, Proximate Composition and Collagen Characteristics

Muscle colour, proximate composition, total collagen, collagen crosslink and proteoglycan concentrations are presented in Table 3. Of the muscle colour coordinates, ‘L’ and ‘b’ values were higher (*p* < 0.001) for C than for P99, P162 and P231, which did not differ; ‘a’ value was lower (*p* < 0.001) for C and P99 (which did not differ) than for P231, which in turn was lower than for P162; ‘*C*’ value was lower (*p* < 0.001) for C and P99 (which did not differ) than for P162 and P231, which did not differ; and ‘H’ value was higher (*p* < 0.001) for C than for P99, which in turn was higher than for P162 and P231, which did not differ. Intramuscular fat concentration was lower (*p* < 0.001) for P99 than for C, P162 and P231, which did not differ. The total collagen concentration did not differ between production systems but the proportion (of total collagen) of soluble collagen was higher (*p* < 0.01) for C and P99 (which did not differ) than for P162 and P231, which did not differ. Collagen crosslink concentration was lower (*p* < 0.05) for C than for P162 and P231 (which did not differ) but similar to P99, which in turn was similar to P162. Production system did not affect muscle proteoglycan concentrations.

### 3.4. Muscle Metabolic Enzyme Activities and Fibre Types

Muscle metabolic enzyme activity and fibre type data are presented in Table 4. When expressed as µmol min^−1^·g^−1^ of tissue, LDH activity was higher (*p* < 0.01) for C, P99 and P162 (which did not differ) than for P231. When expressed on a protein basis, these differences disappeared. Fibre type I was lower (*p* < 0.01) for C and P99 (which did not differ) than for P162 and P231, which did not differ. Fibre type IIX was higher (*p* < 0.05) for C than for P99 and P231 (which did not differ) but similar to P162, which in turn was similar to P231.

### 3.5. Sensory Characteristics

Muscle sensory characteristics data are presented in Table 5. Tenderness was higher (*p* < 0.001) for C and P162 (which did not differ) than for P99 and P231, which did not differ. Juiciness was higher (*p* < 0.01) for P231 than for C and P99 (which did not differ) but similar to P162, which in turn was similar to C and P99. Flavour liking was higher (*p* < 0.01) for C than for P99 and P231 (which did not differ) but similar to P162, which in turn was similar to C and P99. Overall liking was higher (*p* < 0.001) for C than for P99 and P231 (which did not differ) but similar to P162, which in turn was similar to P231. Ease of cutting was higher (*p* < 0.01) for C than for P99 and P231 (which did not differ) but similar to P162, which in turn was similar to C and P99. Cleanness of cut was higher (*p* < 0.01) for C and P162 (which did not differ) than for P99 and P231, which did not differ. Toughness (both during in-bite and eating) was lower (*p* < 0.001) for C and P162 (which did not differ) than for P99 and P231, which did not differ. Juiciness (during in-bite) was higher (*p* < 0.001) for P162 and P231 (which did not differ) than for P99, but similar to C, which in turn was similar to P99. Chewiness was lower (*p* < 0.001) for C than for P99 and P231 (which did not differ) but similar to P162, which in turn was similar to C. Greasiness (both during eating and residual) was higher (*p* < 0.001) for C than for P99, but similar to P162 and P231, which in turn was similar to P99. Pulpiness (both during eating and residual) was higher (*p* < 0.001) for P231 than for P99, but similar to C and P23, which in turn was similar to P99. Dissolubility was higher (*p* < 0.001) for C than for P99 and P231 (which did not differ) but similar to P162, which in turn was similar to P99 and P231. Ease of swallow was higher (*p* < 0.001) for C than for P99 and P231 (which did not differ) but similar to P162, which in turn was similar to P231.

### 3.6. Correlations Between Production Traits, Muscle Biochemical Composition and Sensory Qualities of Beef

The partial correlations between production/carcass traits, and muscle biochemical and sensory traits of beef are summarised in Table 6. Animal age was positively correlated (*p* < 0.01) with temperature at 48 h post-mortem. Average daily gain in the finishing period was negatively correlated with collagen cross link (*p* < 0.05) and tended (*p* < 0.10) to be negatively correlated with proteoglycan concentrations and positively correlated with abnormal flavour. Average daily gain overall was positively correlated (*p* < 0.05, at least) with all muscle colour traits except the ‘a’ value. Both slaughter weight and carcass weight were positively correlated (*p* < 0.05, at least) with temperature at 48 h post-mortem and with muscle ‘a’, ‘b’ and chroma/saturation values. Carcass conformation score was positively correlated (*p* < 0.05, at least) with muscle ‘a’, ‘b’ and chroma/saturation values and negatively correlated (*p* < 0.05) with subcutaneous fat ‘L’ value, IMF and collagen cross link concentrations. Carcass fat score was positively correlated (*p* < 0.05) with subcutaneous fat ‘a’, ‘b’ and chroma/saturation values, with the proportion of muscle fibre type IIA and tended (*p* < 0.10) to be positively correlated with IMF concentration. Carcass fat score was negatively correlated (*p* < 0.05) with subcutaneous fat ‘*H*’ value and muscle moisture concentration and tended (*p* < 0.10) to be negatively correlated with the proportion of muscle fibre type IIX. 

The partial correlations between muscle sensory and biochemical traits of beef are summarised in Table 7. Tenderness tended (*p* < 0.10) to be negatively correlated with collagen cross link concentration. Juiciness was negatively correlated (*p* < 0.05) with ‘*L*’ and ‘*H*’ values but positively correlated (*p* < 0.05) with the ‘*a*’ value and tended (*p* < 0.10) to be positively correlated with the chroma/saturation value. Beef flavour was positively correlated (*p* < 0.05) with IMF concentration and negatively correlated (*p* < 0.05) with ‘*b*’ value and collagen cross link concentration and tended (*p* < 0.10) to be negatively correlated with ‘*L*’ value, ‘*H*’ value and protein concentration. Abnormal flavour was positively correlated (*p* < 0.05) with ‘a’ value and the chroma/saturation value and tended (*p* < 0.10) to be positively correlated with fibre type IIA. Abnormal flavour tended (*p* < 0.10) to be negatively correlated with IMF concentration and ICDH activity. Flavour liking was negatively correlated (*p* < 0.05) with protein and collagen cross link concentrations, tended (*p* < 0.10) to be negatively correlated with proteoglycan concentration, was positively correlated (*p* < 0.05) with ash concentration and tended (*p* < 0.10) to be positively correlated with CS activity. Overall liking was negatively correlated (*p* < 0.05) with proteoglycan concentration, tended (*p* < 0.10) to be negatively correlated with protein concentration, was positively correlated (*p* < 0.05) with ash concentration and tended (*p* < 0.10) to be positively correlated with CS activity. 

## 4. Discussion

In temperate climates, maximising the proportion of grazed grass in the diet of beef cattle generally decreases the cost of production. The primary objective of the present study was to exploit a full grazing season in a suckler bull production system. From a carcass quality perspective, the main concern in this study was meeting the carcass weight and fat classification required in several European markets. From a meat quality perspective, the main concern was whether the increasing age/weight at slaughter as the period at pasture increased would have a negative effect. The slaughter strategies chosen were based on the modification of the more traditional sucker bull beef production (high concentrate feeding indoors to less than 16 months of age) whereby a relatively short grazing period is included and animals are slaughtered to a carcass weight target (C v P99) [2] and then extending the grazing period but slaughtering at a similar fat classification as the traditional system (C, P162, P231). These strategies were achieved with respect to carcass weight, similar for C and P99 and fat classification, similar for C, P162 and P231. The low average carcass fat classification for the P99 bulls reflects the shorter finishing period on concentrate to achieve the target carcass weight. Some lucrative bull beef markets require a carcass fat classification of ≥6 (on a 1–15 scale). Of the 14 carcasses in P99, 7 were in fat class 6 i.e., borderline acceptable (6 were in fat class 3 and 1 in fat class 9), which highlights the dependence of good grass growth and management in maximizing the role of grazed grass in producing “lighter” carcasses within this system. However, from a lean meat production perspective, the lower average fat score, at a similar carcass weight, was reflected in lower fat deposition in the cube roll joint highlighting the positive effect of grazing in this regard. Achieving the current market specification for fat score at 18 months of age is most easily done by using an early maturing sire breed [21]. 

As designed, the increase in the duration of grazing followed by concentrate feeding to meet the fat classification target increased the age at slaughter resulting in a range in mean age of 9 months approximately. The higher growth rate prior to slaughter (finishing period) for the P99 bulls can be explained by the shorter indoor finishing period as an increase in the length of the finishing period results in a progressive decrease in live weight gain [22]. With regard to subcutaneous fat colour, the higher lightness for C, P162 and P231 bulls may be attributed to the higher fat scores of their carcasses compared to the P99 bulls. A similar observation on the relationship between fat classification and fat lightness was made by Mezgebo et al. [4] Based on the review of factors influencing fat colour [23] we hypothesised that fat yellowness would increase as the duration of the grazing period increased. The data do not support this hypothesis. If the fat had become more yellow due to grazing and deposition of carotenoids from the grass [23], it seems the length of the finishing period on concentrates was sufficient to remove this effect.

**Figure 2:**
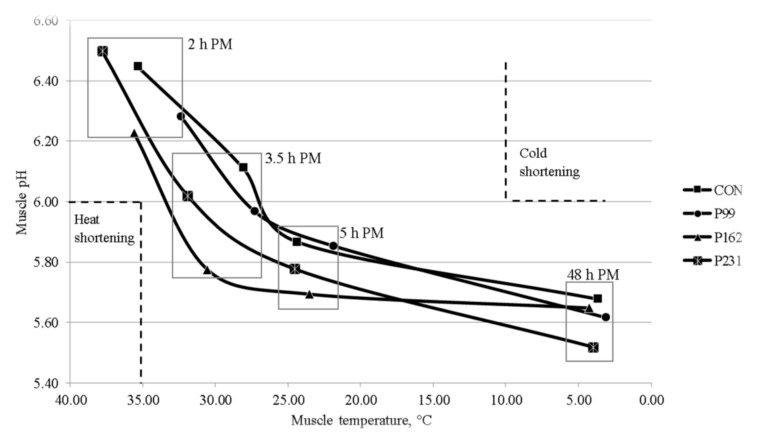
Post-mortem muscle pH relative to muscle temperature for bulls raised on either a concentrate system (C) or pasture-based systems (P) incorporating 99 days (P1), 162 days (P2) or 231 days (P3) of grazing prior to indoor finishing on a concentrate-based diet (*n* = 14/treatment). The zones of heat shortening (temperature ≥ 35 °C at pH = 6) and cold shortening (temperature ≤ 15 °C at pH = 6) are indicated by the dashed lines. Measurements were made at 2, 3.5, 5 and 48 h post-mortem (PM).

The lower fat classification of the P99 bulls may have contributed to the faster post-mortem decline in LT temperature of these bulls compared to the C, P162 and P231 bulls. The post-mortem pH profile is mainly determined by muscle glycogen content at slaughter, which in turn is influenced by pre-slaughter nutrition and stress levels of the animal before and at slaughter [24]. In the present study, the animals were managed to avoid stress-related loss of glycogen mainly by finishing indoors and careful handling during transport and in the lairage. While variations in the pattern of decline in the pH of LT were observed between the groups, none of the carcasses were likely to have been affected by ‘cold shortening’, muscle pH > 6 at muscle temperature < 10 °C [25] or ‘heat shortening’, muscle pH < 6 at muscle temperature >35 °C [26,27] (Figure 2). 

The lower pH at 48 h post-mortem for the P231 bulls than for the C, P99 and P231 bulls suggests that the glycogen level was higher before slaughter in LT from these bulls. However, LT pH values from all groups were within the ‘normal’ pH range of 5.4–5.8 [28] and no carcasses were deemed “dark cutters” by abattoir personnel. Nevertheless, when adjusted for production system effects i.e., partial correlation, pH was negatively correlated with LT lightness (r = −0.45, *p* < 0.001) and ‘hue’ angle (r = −0.41, *p* < 0.01).

The higher redness and colour saturation and lower ‘hue’ angle of LT from P162 and P231 bulls compared to C and P99 bulls, and the higher lightness for the C bulls compared to P99, P162 and P231 bulls, can be explained by the older age at slaughter as muscle tissue becomes redder and darker with increasing slaughter age [29,30]. The lack of effect of slaughter age per se on colour variables when adjusted for production system effects in the present study, however, highlights the confounding effect of other production variations on LT colour. It is likely that increasing the grazing period increased the total amount of exercise engaged in by the animals when at pasture. However, data with respect to exercise and muscle colour in cattle are equivocal [31]. The higher proportion of LT type I fibres (characteristic of red muscle) for the P162 and P231 bulls compared to C and P99 bulls could also contribute to the higher redness. 

Deposition of fat generally increases with increasing age and carcass weight of beef cattle [32,33]. However, in the present study, the higher IMF concentration (and the proportion of fat in the cube roll joint) for C compared to P99 bulls despite being younger at slaughter reflects their higher energy consumption and higher growth rate over the full experimental period as high nutrient/energy intake results in higher fat deposition [32,33,34]. Tat the IMF concentration (and the proportion of fat in the cube roll joint) was similar for P162 and P231, despite the latter being older and having a heavier carcass at slaughter might reflect a residual effect of the longer grazing period since grazing generally results in lower fat deposition [4,35]. According to [33], animal age is one of the main factors that determine the physical and chemical properties of muscle connective tissue as the animal ages, the proportion of heat stable (chemically non-reducible) crosslinks, such as pyridinoline and Ehlichs chromagen, increases [36,37], which in turn leads to a decrease in the proportion of heat soluble collagen [37,38]. On a production system basis, the data from the present study support these reports with regard to collagen solubility. However, the lack of a relationship between slaughter age, slaughter weight or carcass weight and collagen solubility, when adjusted for production system effects, indicates that the maturation of collagen is not due to one dominant influence. Moreover, while the lower collagen solubility in LT from P162 and P231 bulls compared to C and P99 bulls could be hypothesised to be due to the higher pyridinoline cross link concentration (P162 numerically but not significantly, higher than P99), when adjusted for production system effects this relationship was not significant.

As a general trend, as an animal becomes older (especially beyond sexual maturity) its muscle undergoes less glycolytic and more oxidative metabolism [39,40]. The lower activities of the glycolytic enzymes, LDH and PFK (tendency), in LT from the P231 bulls in the present study could therefore be attributed to their older age at slaughter compared to the C, P99 and P162 bulls. However, these three groups had similar LDH and PFK activities in LT despite their differences in age indicating that age is not the only factor influencing the activity of these enzymes. The higher proportion of slow twitch type I oxidative fibres in LT from the P162 and P231 bulls indicates that an increase in age at slaughter can lead to an increase in oxidative fibres as reported by Jurie et al. [39] which in turn can lead to an increase in oxidative metabolism of the muscle. However, there was no statistically significant difference in the activity of the oxidative enzymes ICDH, COX and CS. In addition, the higher proportion of type I oxidative fibres in LT from P162 and P231 bulls compared to P99 bulls could also reflect more physical activity due to the extended grazing period [41,42]. The higher proportion of type IIX (fast twitch glycolytic) fibres in LT from C bulls compared to P99 bulls and generally higher for P162 and P231 bulls than for the P99 bulls can be explained mainly by the longer duration of concentrate finishing as high energy intake favours glycolytic muscle metabolism [41,43]. In the present study, the type IIB (fast twitch glycolytic, data not shown) muscle fibre was identified in only 7 animals (1 in C, 4 in P99, none in P162, 2 in P231 bulls; 1 Charolais and 6 Limousin sired bulls)). This might indicate that expression of Type IIB fibre is breed specific as it was reported to be identified commonly in Blonde d’Aquitaine, a French beef breed [44].

The perception that beef, and bull beef in particular, becomes less tender and less acceptable to the consumer as an animal becomes older is an important contributor to the inclusion of age limits in market specifications for bull beef. For steers and heifers, there appears to be little effect of age on tenderness, at least up to 24 months [45]. The lack of a significant relationship between age and tenderness, when adjusted for production system effects, in the present study indicates that a similar conclusion can be made for bulls. In support of this, Dikeman et al. [46] observed no difference in shear force or sensory tenderness in LT from early maturing bulls as age at slaughter increased from 12 to 24 months. Similarly, a literature review of mainly French production systems indicated that as age at slaughter increased from 12 to 24 months there was little evidence of an increase in shear force or decrease in sensory tenderness in meat from bulls [47]. In Irish studies with bulls of dairy origin, there was no difference in shear force or sensory tenderness of LT from production systems similar to C, P99 and P162 where the dairy bulls were 15, 19 and 22 months of age at slaughter, respectively [48]. It is difficult, however, in many studies to separate the effects of age per se from other production factors, which might contribute to differences in beef tenderness. In a study with similar bulls as those used in this study, when averaged across three different production systems, an increase in age from 14.5 to 21.2 months did not affect LT tenderness, however, there was an effect of production system per se [4]. With regard to the eating quality of beef, the differences in sensory characteristics between the treatments in the present study likely reflect the combined and possibly interactive effect of the variations in pre-slaughter growth rate, IMF concentration, muscle fibre distribution and collagen crosslinks. The lower tenderness, flavour liking and overall liking scores for the P231 bulls compared to the C bulls could mainly be attributed to their higher collagen cross-link content and altered fibre distribution since the IMF concentration was similar. Similarly, the lower tenderness and flavour scores for the P99 bulls compared to the C bulls could be related to the lower IMF concentration since muscle structure was not different. The favourable juiciness scores for both P162 and P231 bulls and the higher juiciness scores for the P231 bulls compared to the P99 bulls can possibly be related to their older age at slaughter as beef from older and fatter animals is perceived to be juicier than beef from younger and leaner animals [7,8].

## 5. Conclusions

This study showed that extending the grazing period of pasture-based systems to 162 and 231 days prior to finishing indoors on a high energy concentrate diet, and the associated increase in age at slaughter, produced bulls with a similar carcass fat cover and IMF concentration to the intensively concentrate fed bulls. With regard to eating quality, extending the grazing period to 162 days resulted in beef with a sensory quality (tenderness, flavour liking and overall liking) similar to beef from the intensively concentrate fed bulls. However, a further increase in the grazing period (i.e., to 231 days) led to the production of beef with a lower tenderness, flavour liking and overall liking scores and higher juiciness values compared to the intensively concentrate fed bulls. These sensory attributes were also adversely affected by a shorter finishing period on concentrates (71 v. 120 days) after grazing. The impact of post slaughter management of the latter carcasses, such as electrical stimulation or aitch bone hanging, merit investigation. The data also indicate that age per se without reference to the production system is not an appropriate market specification from a meat quality perspective.

## Figures and Tables

**Table 1:** Production and carcass traits of bulls raised on either a concentrate-based system (C) or pasture-based systems incorporating 99 days (P99), 162 days (P162) or 231 days (P231) grazing prior to indoor finishing on a concentrate-based diet.

	C	P99	P162	P231	s.e.m	Significance
Number of animals	14	14	14	14		
Finishing period (days) ^1^	201	71	120	121		
Age at slaughter (months)	15.0 ^a^	18.3 ^b^	22.0 ^c^	24.5 ^d^	0.200	***
ADG ^2^ finishing (kg·day^−1^)	1.50 ^ab^	2.04 ^c^	1.34 ^a^	1.65 ^b^	0.081	***
ADG overall (kg·day^−1^)	1.58 ^c^	1.10 ^b^	1.10 ^a^	1.06 ^a^	0.027	***
Slaughter weight (kg)	667.0 ^a^	692.6 ^a^	774.6 ^b^	829.6 ^c^	13.25	***
Carcass weight (kg)	378.9 ^a^	386.9 ^a^	437.6 ^b^	474.7 ^c^	8.09	***
Conformation ^3^	9.86	9.71	9.14	10.36	0.318	0.07
Fat score ^4^	8.36 ^b^	6.64 ^a^	8.21 ^b^	8.85 ^b^	0.289	***
Fat colour ^5^						
‘L’	68.63 ^b^	64.47 ^a^	67.06 ^b^	68.12 ^b^	0.614	***
‘a’	8.75	9.32	9.68	9.63	0.445	0.44
‘b’	15.57	15.38	15.60	15.79	0.351	0.88
‘C’	17.91	17.99	18.37	18.53	0.493	0.78
‘H’	61.11	58.87	58.34	58.77	0.888	0.13
Rib joint (kg)	10.6 ^a^	10.2 ^a^	12.3 ^b^	13.2 ^b^	0.25	***
*Longissimus thoracis* (kg)	2.04 ^a^	2.27 ^ab^	2.45 ^b^	2.50 ^b^	0.089	**
Area (cm^2^)	953 ^a^	1077 ^b^	1119 ^b^	1075 ^b^	40.4	*
Rib joint composition (g/kg)						
Total lean	667.5 ^a^	708.2 ^b^	652.0 ^a^	647.8 ^a^	8.92	***
Fat	139.5 ^b^	86.7 ^a^	146.6 ^b^	145.3 ^b^	7.38	***
Bone	193.0	205.0	201.5	206.9	4.40	0.13

(*n* = 14/treatment). ^1^ Days on *ad libitum* concentrates prior to slaughter; ^2^ Average daily live weight gain; ^3^ Conformation classes: E^+^ (highest) to P^−^ (lowest), (E^+^ is 15); ^4^ Fat score classes 5^+^ (highest) to 1^−^ (lowest), (5^+^ is 15); ^5^ Subcutaneous fat colour: ‘L’ = lightness, 0 (black) to 100 (white); ‘a’ = redness, +a (red) to −a (green); ‘b’ = yellowness, +b (yellow) to −b (blue); ‘C’ = chroma, higher ‘C’ values higher colour saturation; ‘H’ = hue, 0/360° is red, 90° is yellow, 180° is green and 270° is blue colour; ^a,b,c,d^ means within rows, assigned different superscripts differ significantly (*p* < 0.05); s.e.m: standard error of the mean; * *p* < 0.05; ** *p* < 0.01; *** *p* < 0.001.

**Table 2:** Post-mortem pH and temperature of longissimus thoracis muscle from bulls raised on either a concentrate-based system (C) or pasture-based systems incorporating 99 days (P99), 162 days (P162) or 231 days (P231) grazing prior to indoor finishing on a concentrate-based diet.

	System	Time, Post-Mortem (h)			s.e.m.	Significance		
		2	3.5	5		System	Time	System × Time
pH	C	6.45 ^b^	6.11 ^b^	5.87 ^b^	0.055	**	***	***
	P99	6.28 ^a^	5.97 ^b^	5.85 ^b^				
	P162	6.23 ^a^	5.78 ^a^	5.69 ^a^				
	P231	6.50 ^b^	6.02 ^b^	5.78 ^a,b^				
Temperature	C	35.32 ^b^	28.09 ^a^	24.38 ^b^	0.424	***	***	***
	P99	32.39 ^a^	27.30 ^a^	21.86 ^a^				
	P162	35.62 ^b^	30.57 ^b^	23.52 ^b^				
	P231	37.78 ^c^	31.90 ^c^	24.56 ^b^				

(*n* = 14/treatment). ^a,b,c^ means within columns for pH and temperature, assigned different superscripts, differ significantly (*p* < 0.05); s.e.m: standard error of the mean; ** *p* < 0.01; *** *p* < 0.001.

**Table 3:** pH at 48h post-mortem, colour, proximate composition and connective tissue characteristics of longissimus thoracis muscle from bulls raised on either a concentrate-based system (C) or pasture-based systems incorporating 99 days (P99), 162 days (P162) or 231 days (P231) grazing prior to indoor finishing on a concentrate-based diet.

	C	P99	P162	P231	s.e.m	Significance
pH Muscle colour ^1^	5.68 ^a^	5.62 ^b^	5.65 ^b^	5.52 ^c^	0.018	***
‘L’	32.77 ^b^	28.25 ^a^	27.18 ^a^	28.98 ^a^	0.693	***
‘a’	20.47 ^a^	21.15 ^a^	23.63 ^c^	22.70 ^b^	0.317	***
‘b’	13.93 ^b^	12.91 ^a^	13.32 ^a^	13.15 ^a^	0.216	**
‘C’	24.78 ^a^	24.78 ^a^	27.14 ^b^	26.24 ^b^	0.334	***
‘H’	34.23 ^c^	31.40 ^b^	29.42 ^a^	30.12 ^a^	0.423	***
Proximate composition (g/kg)						
Fat	23.23 ^b^	10.18 ^a^	20.77 ^b^	19.85 ^b^	1.694	***
Moisture	747.1	748.7	743.9	739.5	3.66	0.31
Protein	228.5	230.7	232.7	228.1	2.43	0.52
Ash	11.19	11.31	10.94	11.29	0.268	0.74
Collagen content						
Total collagen (mg g^−1^)	3.86	3.87	3.92	3.87	0.127	0.99
Soluble collagen (%)	9.38 ^b^	9.44 ^b^	7.09 ^a^	6.86 ^a^	0.653	**
Collagen cross-links ^2^						
(nM PYD g^−1^ DM)	18.52 ^a^	19.56 ^a,b^	20.59 ^b,c^	21.28 ^c^	0.628	*
Proteoglycan ^3^						
(µg C4S-GAGs g^−1^ DM)	173.9	171.4	163.9	181.7	9.41	0.62

(*n* = 14/treatment). ^1^ Muscle colour: ‘L’ = lightness, 0 (black) to 100 (white); ‘a’ = redness, +a (red) to −a (green); ‘b’ = yellowness, +b (yellow) to −b (blue); ‘C’ = chroma, higher ‘C’ values higher colour saturation; ‘H’ = hue, 0/360° is red, 90° is yellow, 180° is green and 270° is blue colour. ^2^ nM of pyridinoline (PYD) cross-links per g of dry matter. ^3^ µg of chondroitine-4-sulfate (C4S)-glycosaminoglycans (GAGs) per g of dry matter. ^a,b,c^ means within rows, assigned different superscripts differ significantly (*p* < 0.05). s.e.m: standard error of means; * *p* < 0.05; ** *p* < 0.01; *** *p* < 0.001.

**Table 4:** Metabolic enzyme activity and fibre type characteristics of longissimus thoracis muscle from bulls raised on either a concentrate-based system (C) or pasture-based systems incorporating 99 days (P99), 162 days (P162) or 231 days (P231) grazing prior to indoor finishing on a concentrate-based diet.

	C	P99	P162	P231	s.e.m	Significance
Metabolic enzyme activity ^1^						
µmol min^−1^ g^−1^ of tissue						
LDH	998.6 ^b^	968.7 ^b^	992.2 ^b^	885.1 ^a^	24.17	**
PFK	111.7	112.4	102.0	93.7	5.89	0.09
ICDH	1.01	1.02	1.11	1.20	0.068	0.17
COX	15.11	15.21	14.23	15.95	1.315	0.83
CS	5.34	4.58	3.87	4.92	0.463	0.16
µmol min^−1^·g^−1^ of protein						
LDH	5007.3	5477.8	5122.6	4597.7	275.92	0.17
PFK	559.3	636.2	526.7	486.2	41.59	0.08
ICDH	5.12	5.68	5.74	6.23	0.409	0.31
COX	75.70	87.06	73.17	81.84	7.985	0.61
CS	27.09	26.23	19.79	25.53	2.823	0.26
Protein (mg/g of tissue)	199.6	185.7	194.4	193.3	5.04	0.28
Muscle fibre types (%) ^2^						
I	18.48 ^a^	17.08 ^a^	22.46 ^b^	23.14 ^b^	1.45	**
IIA	38.62	46.77	37.71	43.04	3.05	0.14
IIX	44.14 ^c^	29.83 ^a^	39.83 ^b,c^	33.33 ^a,b^	3.53	*

(*n* = 14/treatment). ^1^ LDH: lactate dehydrogenase; PFK: phosphofructokinase; ICDH: isocitrate dehydrogenase; COX: cytochrome c oxidase; CS: citrate synthase. ^2^ I: oxidative, IIA: oxido-glycolytic, IIX: glycolytic. ^a,b,c^ means within rows, assigned different superscripts differ significantly (*p* < 0.05). s.e.m: standard error of means; * *p* < 0.05; ** *p* < 0.01.

**Table 5:** Sensory characteristics of longissimus thoracis muscle from bulls raised on either a concentrate-based system (C) or pasture-based systems (P) incorporating 99 days (P1), 162 days (P2) or 231 days (P3) of grazing period prior to indoor finishing on a concentrate-based diet.

	C	P99	P162	P231	s.e.m	Significance
Attribute, scale 1 (least)–8 (most)						
Tenderness	4.63 ^b^	4.20 ^a^	4.60 ^b^	4.25 ^a^	0.090	***
Juiciness	4.83 ^a^	4.81 ^a^	4.95 ^ab^	5.09 ^b^	0.065	**
Beefy flavour	4.55	4.51	4.52	4.52	0.059	0.96
Abnormal flavour	2.30	2.42	2.41	2.48	0.071	0.35
Flavour liking	5.46 ^b^	5.10 ^a^	5.19 ^ab^	5.12 ^a^	0.078	**
Overall liking	5.03 ^c^	4.59 ^a^	4.90 ^bc^	4.67 ^ab^	0.076	***
Specific sensory indicators, scale 0 (nil)–100 (extreme)						
*On-cut*						
Ease of cutting	53.51 ^b^	46.72 ^a^	50.04 ^ab^	47.01 ^a^	1.401	**
Cleanness of cut	56.64 ^b^	53.88 ^a^	58.46 ^b^	54.44 ^a^	1.266	*
*In-bite*						
Toughness	45.47 ^a^	54.89 ^b^	47.43 ^a^	53.22 ^b^	1.357	***
Crispness	24.34	25.36	25.88	26.95	1.121	0.42
Juiciness	46.74 ^ab^	44.19 ^a^	48.22 ^b^	49.82 ^b^	0.996	***
Sponginess	28.58	25.51	27.46	26.93	0.829	0.07
*Eating*						
Toughness	44.89 ^a^	53.50 ^b^	48.10 ^a^	52.95 ^b^	1.322	***
Moisture	48.11	44.97	48.13	48.59	1.059	0.06
Chewiness	42.58 ^a^	48.98 ^b^	45.39 ^ab^	50.26 ^b^	1.456	***
Greasiness	19.06 ^b^	15.74 ^a^	16.97 ^ab^	16.92 ^ab^	0.794	*
Fibres	42.64	46.11	44.52	45.99	1.051	0.07
Gristle	6.43	6.16	7.22	7.22	0.811	0.72
Pulpy	52.70 ^ab^	50.16 ^a^	53.93 ^ab^	54.1 ^b^	1.049	*
Dissolubility	49.63 ^b^	42.97 ^a^	46.94 ^ab^	43.16 ^a^	1.307	***
*Residual*						
Greasiness	18.44 ^b^	15.42 ^a^	16.55 ^ab^	16.48 ^ab^	0.770	*
Ease of swallow	59.48 ^c^	52.54 ^a^	57.12 ^bc^	53.86 ^ab^	1.220	***
Pulpy	51.89 ^ab^	48.18 ^a^	52.03 ^ab^	53.20 ^b^	1.137	*
Particles	48.94	52.45	52.09	50.93	1.013	0.06
Mouthfeel	52.23	49.82	52.64	53.21	0.984	0.08

(*n* = 14/treatment). ^a,b,c^ means within rows, assigned different superscripts differ significantly (*p* < 0.05). s.e.m: standard error of means; * *p* < 0.05; ** *p* < 0.01; *** *p* < 0.001.

**Table 6:** Partial correlations (r) between animal/carcass traits and longissimus muscle biochemical/sensory traits ^1^.

Trait	Age	ADGi	ADGo	Weight	Carcass Weight	Conformation	Fat Score
pH 48 h	0.045	−0.221	−0.230 ^+^	−0.149	−0.183	−0.199	0.118
Temperature 48 h	0.362 **	0.182	0.217	0.507 ***	0.510 ***	0.210	0.110
Lightness	−0.152	0.196	0.391 **	0.153	0.220	0.138	−0.178
Redness	0.184	0.072	0.202	0.313 *	0.322 *	0.265 *	0.116
Yellowness	−0.036	0.234 ^+^	0.468 ***	0.323 *	0.387 **	0.279 *	−0.109
Saturation	0.133	0.142	0.325 *	0.364 *	0.393 **	0.309 *	0.057
Hue	−0.201	0.177	0.300 *	0.063	0.116	0.050	−0.211
Fat lightness	0.117	−0.145	0.032	0.077	0.033	−0.324 *	0.112
Fat redness	0.033	0.108	0.216	0.251 ^+^	0.218	0.160	0.430 **
Fat yellowness	0.058	0.031	0.164	0.222	0.143	−0.044	0.411 **
Fat saturation	0.049	0.069	0.197	0.247 ^+^	0.185	0.048	0.450 ***
Fat Hue	−0.025	−0.132	−0.199	−0.219	−0.226	−0.255 ^+^	−0.311 *
Moisture	0.158	−0.003	−0.264 *	−0.168	−0.158	−0.036	−0.346 *
Intramuscular fat	0.229	−0.106	−0.202	−0.111	−0.162	−0.276 *	0.252 ^+^
Protein	−0.273	0.085	0.232 ^+^	0.042	0.063	0.181	0.052
Ash	0.009	0.150	0.123	0.055	0.148	0.209	−0.239 ^+^
Soluble collagen	0.002	−0.003	−0.094	0.006	0.051	−0.008	−0.029
Insoluble collagen	0.104	0.185	−0.004	0.125	0.033	−0.196	−0.006
Total collagen	0.099	0.171	−0.026	0.118	0.044	−0.183	−0.014
Soluble collagen %	−0.057	−0.070	−0.066	−0.029	0.046	0.066	−0.028
PYDCL	−0.126	−0.249 ^+^	−0.066	−0.119	−0.170	−0.286 *	0.019
GAGS	−0.027	−0.287 *	−0.062	−0.144	−0.108	−0.176	−0.138
ICDH	0.150	−0.189	−0.107	−0.003	−0.003	−0.140	−0.004
LDH	0.003	−0.119	−0.148	−0.161	−0.141	0.023	−0.067
PFK	0.081	−0.028	−0.025	0.026	0.038	0.074	−0.069
COX	0.185	−0.107	−0.024	0.084	0.069	−0.059	0.128
CS	0.093	0.013	−0.075	−0.018	−0.026	−0.054	0.128
IIA	0.134	0.053	0.158	0.201	0.164	0.064	0.449 ***
I	0.114	−0.146	0.046	0.048	0.061	0.034	0.117
IIX	−0.170	0.084	0.068	−0.013	0.070	0.084	−0.264 ^+^
Tenderness	−0.161	−0.199	−0.132	−0.135	−0.148	0.056	0.082
Juiciness	0.014	−0.081	0.099	0.156	0.115	0.209	0.071
Beef flavour	0.188	−0.050	−0.237 ^+^	−0.137	−0.171	−0.064	0.098
Abnormal Flavour	−0.031	0.255 ^+^	0.144	0.250 ^+^	0.219	0.104	0.133
Flavour liking	0.108	−0.094	−0.153	−0.156	−0.129	−0.037	−0.123
Overall liking	0.101	−0.115	−0.168	−0.066	−0.083	−0.022	−0.122

^1^ PYDCL: Collagen cross-link; GAGS: proteoglycans; ICDH: isocitrate dehydrogenase; LDH: lactate dehydrogenase; PFK: phosphofructokinase; COX: cytochrome *c* oxidase; CS: citrate synthase; IIA, I, IIX: muscle fibre types. + *p* < 0.10; * *p* < 0.05; ** *p* < 0.01; *** *p* < 0.001.

**Table 7:** Partial correlations (r) between longissimus muscle biochemical and sensory traits ^1^.

Trait	Tenderness	Juiciness	Beef Flavour	Abnormal Flavour	Flavour Liking	Overall Liking
pH 48 h	−0.144	0.031	0.063	0.082	−0.076	−0.057
Temperature 48 h	−0.074	0.241 ^+^	−0.085	0.230	−0.211	−0.111
Lightness	−0.093	−0.403 **	−0.236 ^+^	−0.156	−0.080	−0.189
Redness	0.081	0.374 **	−0.054	0.305 *	−0.150	−0.031
Yellowness	−0.049	−0.189	−0.276 *	0.048	−0.162	−0.189
Saturation	0.045	0.231 ^+^	−0.141	0.270 *	−0.184	−0.096
Hue	−0.138	−0.520 ***	−0.242 ^+^	−0.195	−0.052	−0.181
Moisture	−0.166	−0.183	0.120	0.018	0.048	−0.031
Intramuscular fat	−0.098	−0.124	0.349 *	−0.250 ^+^	0.186	0.093
Protein	−0.076	−0.037	−0.258 ^+^	0.178	−0.263 *	−0.230 ^+^
Ash	0.155	−0.088	0.133	−0.184	0.309 *	0.273 *
Soluble collagen	0.014	−0.019	−0.081	0.090	−0.119	−0.097
Insoluble collagen	−0.132	−0.107	−0.012	−0.025	0.032	−0.025
Total collagen	−0.120	−0.102	−0.029	−0.004	0.004	−0.044
Soluble collagen %	0.074	0.016	−0.082	0.091	−0.124	−0.074
PYDCL	−0.248 ^+^	−0.029	−0.279 *	0.042	−0.268 *	−0.299 *
GAGS	0.043	0.053	−0.096	0.075	−0.233 ^+^	−0.022
ICDH	0.121	0.130	0.054	−0.241 ^+^	0.211	0.220
LDH	0.207	0.103	−0.116	0.109	0.030	0.141
PFK	0.139	0.134	−0.101	0.142	−0.008	0.078
COX	0.201	0.111	0.068	−0.047	0.105	0.216
CS	0.164	−0.009	0.049	−0.190	0.259 ^+^	0.250 ^+^
IIA	0.099	0.131	−0.082	0.259 ^+^	−0.142	−0.054
I	0.109	0.197	0.090	−0.139	0.071	0.098
IIX	−0.056	0.041	−0.027	−0.059	0.095	0.078

^1^ PYDCL: Collagen cross-link; GAGS: proteoglycans; ICDH: isocitrate dehydrogenase; LDH: lactate dehydrogenase; PFK: phosphofructokinase; COX: cytochrome *c* oxidase; CS: citrate synthase; IIA, I, IIX: muscle fibre types. + *p* < 0.10; * *p* < 0.05; ** *p* < 0.01; *** *p* < 0.001.
